# Translating Resuscitation Guidelines into Practice: Health Care Provider Attitudes, Preferences and Beliefs Regarding Pediatric Fluid Resuscitation Performance

**DOI:** 10.1371/journal.pone.0058282

**Published:** 2013-03-12

**Authors:** Melissa J. Parker, Asmaa Manan

**Affiliations:** 1 Department of Pediatrics, McMaster Children's Hospital and McMaster, University, Hamilton, Ontario, Canada; 2 Division of Emergency Medicine, Department of Pediatrics, The Hospital for Sick Children and The University of Toronto, Toronto, Ontario, Canada; Nottingham University, United Kingdom

## Abstract

**Introduction:**

Children who require fluid resuscitation for the treatment of shock present to tertiary and non-tertiary medical settings. While timely fluid therapy improves survival odds, guidelines are poorly translated into clinical practice. The objective of this study was to characterize the attitudes, preferences and beliefs of health care providers working in acute care settings regarding pediatric fluid resuscitation performance.

**Methods:**

A single-centre survey study was conducted at McMaster Children's Hospital from January to May, 2012. The sampling frame (n = 115) included nursing staff, physician staff and subspecialty trainees working in Pediatric Emergency Medicine (PEM) or Pediatric Critical Care Medicine (PCCM). A self-administered questionnaire was developed and assessed for face validity prior to distribution. Eligible participants were invited at 0, 2, and 4 weeks to complete a web-based version of the survey. A follow-up survey administration phase was conducted to improve the response rate.

**Results:**

Response rate was 72.2% (83/115), with 83% (68/82) self-identifying as nursing staff and 61% (50/82) as PCCM providers. Resuscitation experience, frequency of shock management, and years in specialty, were similar between PCCM and PEM responders. Physicians and nurses had differing opinions regarding the most effective method to achieve rapid fluid resuscitation in young children presenting in shock (p<0.001). Disagreement also existed regarding the age and size of patients in whom rapid infuser devices, such as the Level-1 Rapid Infuser, should be used (p<0.001). Providers endorsed a number of potential concerns related to the use of rapid infuser devices in children, and only 14% of physicians and 55% of nursing staff felt that they had received adequate training in the use of such devices (p = 0.005).

**Conclusions:**

There is a lack of consensus among health care providers regarding how pediatric fluid resuscitation guidelines should be operationalized, supporting a need for further work to define best practices.

## Introduction

Shock is a life-threatening condition for infants and children that requires immediate resuscitative intervention. [1]-[5] While the actual incidence of pediatric shock is unknown, it is a worldwide problem, with causes including severe dehydration, bleeding, infection and allergy. With the exception of cardiogenic shock, circulatory instability generally results from conditions of impaired preload due to a state of relative or absolute intravascular hypovolemia. Acute intravascular volume loading increases end diastolic pressure, which in keeping with Frank-Starling principles improves stroke volume and cardiac output. [6] Current resuscitation guidelines require the timely performance of intravascular fluid administration as a critical initial pediatric shock management as this has been shown to improve survival odds. [7]–[9]

Recent reports have led to questions about the role of aggressive fluid resuscitation in pediatric septic shock. [10], [11] However it is imperative to note that these findings relate to children in the developing country setting where malnutrition, malaria, and anemia are common if not endemic. Such factors likely have an important influence on septic shock physiology including the response to fluid therapy. [12]–[15] As such, it is far from clear that these results should be extrapolated to children with septic shock in the developed country setting, or children with other forms of shock residing anywhere. Fluid administration therefore remains a ‘cornerstone’ of initial pediatric shock management.

Translating current fluid resuscitation guidelines into practice remains a challenge. Large volumes of fluid may be required for the effective resuscitation of septic shock, including volumes of up to 100 to 200 mL/kg or more in the first hour. [16], [17] The American College of Critical Care Medicine (ACCM) guideline for the management of pediatric and neonatal septic shock calls for the administration of up to 60 mL/kg of isotonic fluid within the first 15 minutes of resuscitation. [2] While recommended benchmarks are often not achieved, [8], [18], [19] little is known about barriers to optimal pediatric fluid resuscitation performance.

**Figure 1:**
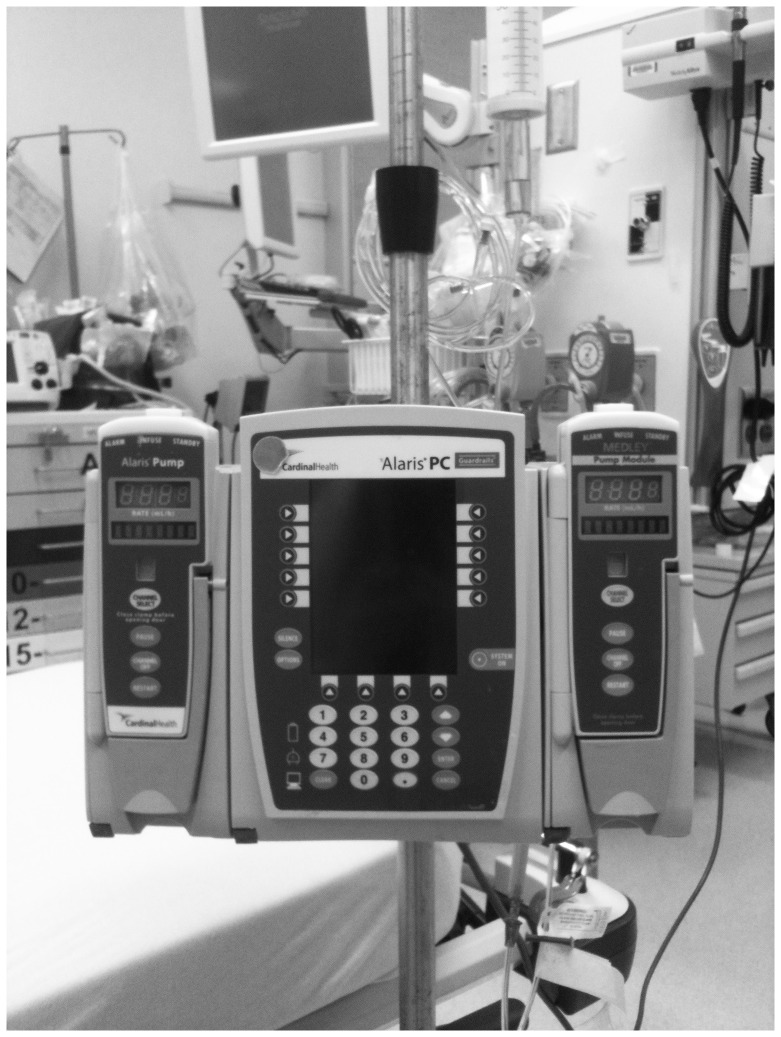
Regular Infusion Pump. Typical bedside infusion pump used for the administration of intravenous fluids, blood products, and medications. Found in most healthcare settings. Maximum fluid infusion rate of 999 mL/hr.

**Figure 2:**
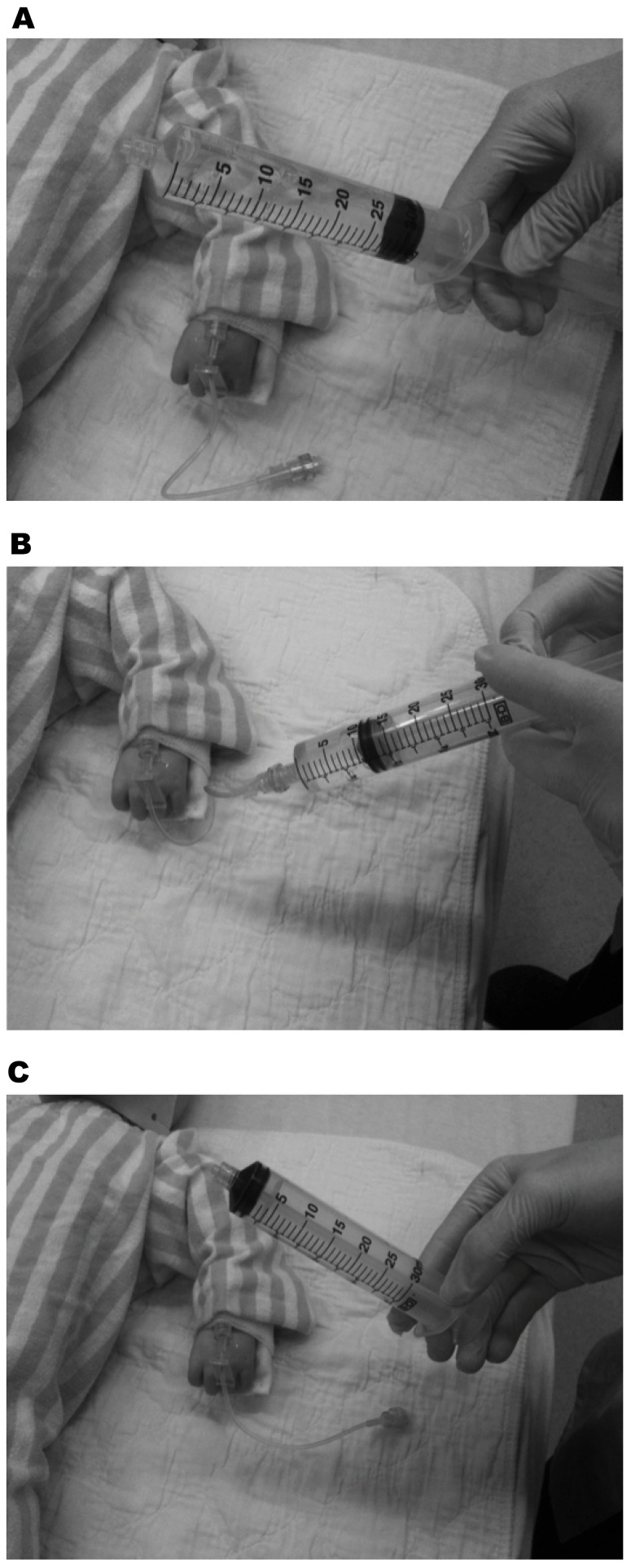
Syringes-in-sequence (Disconnect-Reconnect) method of performing manual fluid resuscitation using syringes. This method of fluid administration involves at least two healthcare providers. One or more health care providers urgently prepare fluid-filled syringes with the isotonic fluid of choice, while another provider administers the fluid to the patient using the syringes as illustrated in the figure. A. Provider takes a fluid-filled syringe prepared by a colleague. B. Provider connects the fluid-filled syringe to the IV extension tubing and rapidly administers the fluid by depressing the syringe plunger. C. Provider disconnects the empty syringe and disposes of it. Steps A through C are repeated as quickly as possible until the desired volume of fluid has been administered. This method of fluid administration is not sophisticated, but is commonly practiced in our experience.

**Figure 3:**
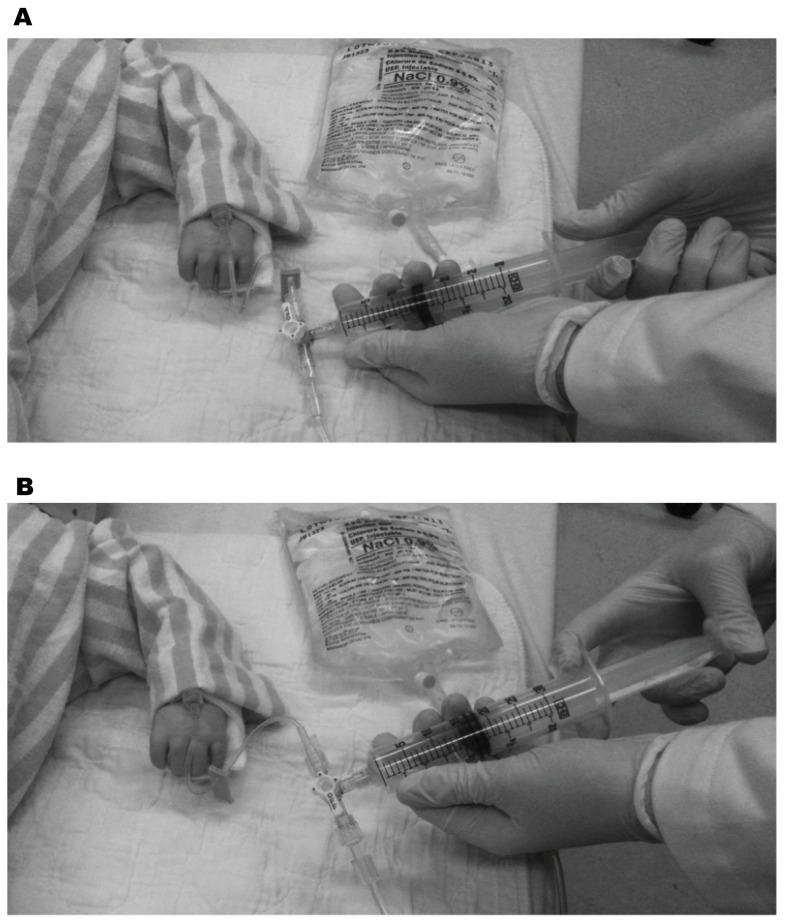
Single syringe (Push-pull) method of performing manual fluid resuscitation using a syringe. This method of fluid administration involves a single healthcare provider. A triple stopcock and IV tubing are required. A. The triple stopcock is toggled to the OFF position to the patient. The provider withdraws fluid from the fluid bag into the syringe by pulling back the syringe plunger. B. The provider then toggles the triple stopcock to the OFF position to the IV fluid bag (ON to the patient). The syringe plunger is then depressed resulting in administration of the fluid within the syringe to the patient. Steps A and B are repeated as quickly as possible until the desired volume of fluid has been administered.

**Figure 4:**
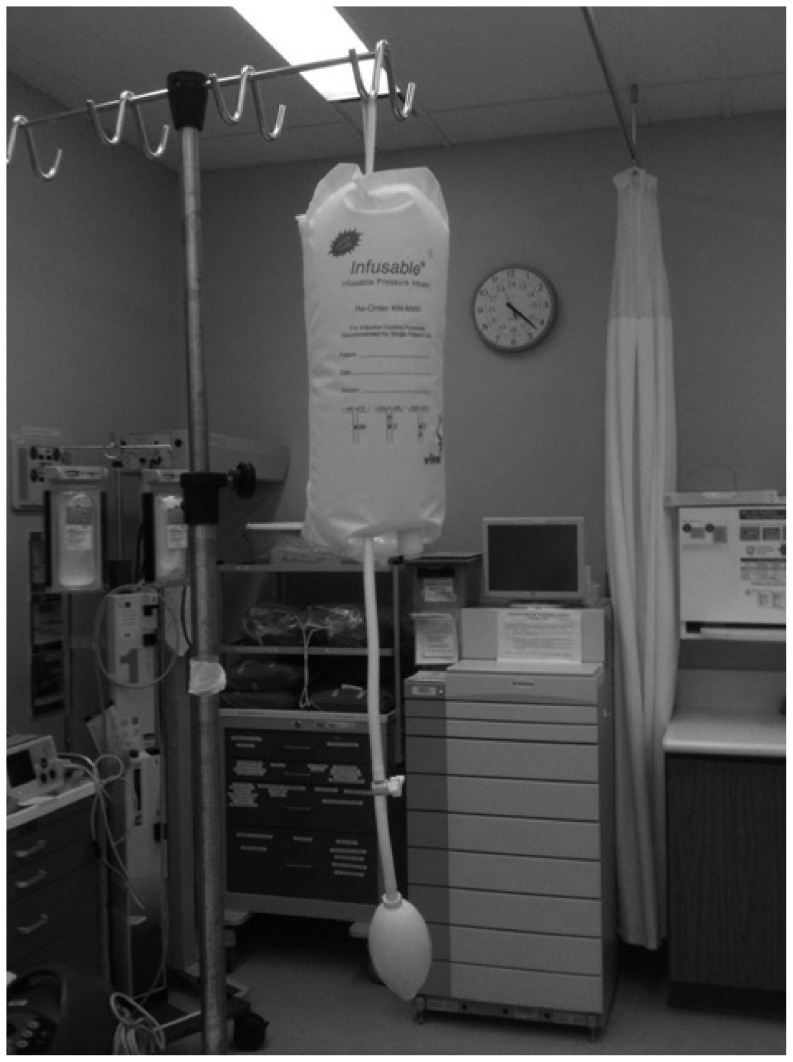
Pressure Bag support. A bag of isotonic fluid is placed within a pressure bag that is manually inflated by a single provider using a pump. Fluid flow rate to the patient is increased due to an increase in the pressure gradient across the intravenous catheter.

**Figure 5:**
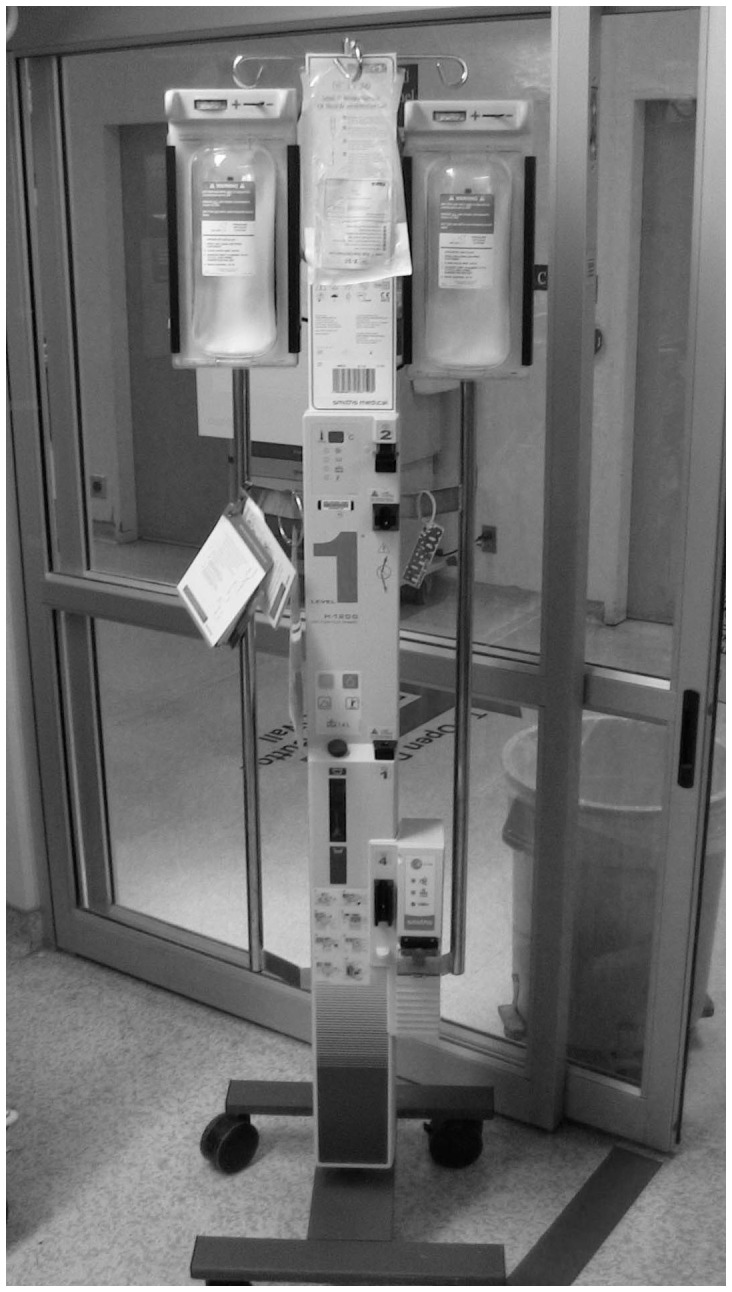
Rapid Infuser Device. Smiths Medical Level 1 H-1200 Fast Flow Fluid Warmer. Up to two bags of isotonic fluid (or blood products) can be placed within the chambers of the device. Pressure around the bags of fluid is mechanically generated leading to a high and consistent pressure of approximately 300 mm Hg. Fluid flow rates of up to 500 mL/min can be achieved. The device does not allow for adjustment of fluid flow rate.

It is important to consider that there are practical and technical challenges associated with simply getting the fluid into the patient when this is emergently required. While the impact of intravenous (IV) catheter [20]–[23] and tubing set [24], [25] properties on fluid flow rates have been studied extensively, provider-level factors have not. To date, a single study has evaluated fluid resuscitation efficiency in pediatric patients in the non-operative setting. [26] A traditional infusion pump (Figure 1), gravity flow, manual syringe techniques (Figures 2 and 3), pressure bag support (Figure 4), or a rapid infuser device (Figure 5) may be used to deliver fluid therapy. Traditional infusion pumps are capable of administering fluid at a maximum rate of 999 mL/hour, which is inadequate for the purposes of resuscitation. In contrast, rapid infuser devices such as the Level 1 (Level 1 H-1200 Fast Flow Fluid Warmer), trademark of Smiths Medical family of companies (Smiths Medical ASD, Inc., Rockland, MA, USA), [27] can administer fluid at high flow rates, however such devices are costly, require operator training, and may not be readily available.

It has been our observation in the non-operative clinical setting that health care providers use a variety of fluid administration techniques in the resuscitation of pediatric patients with decompensated shock – a recognized medical emergency. Given a lack of specific direction in current guidelines as to how fluid administration is best accomplished, we sought to evaluate the attitudes and beliefs of health care providers working in acute care areas regarding pediatric fluid resuscitation performance. We hypothesized that differing attitudes and beliefs would exist among providers suggesting a need for further research to define best practices.

## Materials and Methods

### Ethics Statement

The Faculty of Health Sciences/Hamilton Health Sciences Research Ethics Board approved this study and all procedures were conducted in accordance with the Tri-council Policy Statement: Ethical conduct for research involving humans. [28] A letter of information/consent was provided to all individuals invited to participate in the study. Written consent was not obtained from Phase 1 participants who completed the on-line version of the survey, as this was impracticable. Written consent was obtained and kept on file for Phase 2 study participants who were directly approached and completed the tablet-based version of the survey. Consent procedures were conducted according to the recommendations/requirements of our local Research Ethics Board.

### Study Participants

This single center survey study was conducted at McMaster University Medical Centre (Hamilton, Canada) between January and May 2012. Our sampling frame included all staff nurses (full time, part time and occasional staff), staff physicians (full time, part time and occasional staff), and subspecialty physician trainees working in the Pediatric Emergency Department (PED) or Pediatric Critical Care Unit (PCCU) of McMaster Children's Hospital (n = 115). We chose to survey providers working in the PED and PCCU settings specifically because this group would be expected to be most likely involved in performing pediatric resuscitations. The sampling frame was developed based on information provided by key administrative staff with knowledge of and access to nursing and physician staff lists for these areas. Excluded from participation were medical students and resident trainees other than those pursuing subspecialty training in either Pediatric Critical Care or Pediatric Emergency Medicine. A good command of English was required to participate.

### Survey Development

The survey was developed by the investigators to fulfill the study objectives. Questions elicited demographic information about participants and solicited their attitudes, experiences and beliefs regarding the performance of emergency fluid resuscitation for shock in children of different ages/sizes using different equipment and techniques. Questions also explored attitudes and beliefs concerning safety considerations. A draft version of the questionnaire was assessed for face validity in 5 subjects, who provided verbal and written feedback to the investigators. Comments and suggestions were reviewed and incorporated into the final version of the questionnaire. [29] The finalized version of the survey permitted participants to skip over questions for which they did not wish to provide a response.

### Survey Administration

The protocol involved administration of the survey in two distinct phases. In the first phase, eligible participants were invited via email to complete a web-based (Survey Monkey) version of the survey at 0, 2, and 4 weeks. Participants were instructed to complete the survey only once. A chance to win a $25 coffee card was advertised as an incentive. The protocol included provisions to proceed with a second phase of survey distribution if a response rate of at least 70% had not been achieved by 1 month following the final email inviting study participation.

In the second phase of survey administration, an email was sent to all individuals in the sampling frame alerting them that a research assistant would be visiting the PED and PCCU over a 2-week period to offer a final opportunity to complete the survey. A research assistant visited the PED and PCCU daily and made contact with individuals who appeared available to be approached. Eligible and consenting participants who had not previously completed the survey were presented with an Apple iPad2 tablet device (32 GB with Wi-Fi, Apple model A1395), trademark of Apple Inc. (Apple Inc., Cupertino, CA, USA), to facilitate completion of the survey. The tablet-based version of the survey used during this phase was created using, iSURVEY (iSurveySoft, Wellington, NZ).

### Study Outcomes, Data Management, and Statistical Methods

The primary outcome of this study was participant self-reported attitudes and beliefs regarding pediatric fluid resuscitation practices. As per our a priori plan, we also evaluated the potential impact of practice location (PED vs. PCCU) and profession (physician vs. nursing staff) on survey responses. The sample size was dictated by the number of eligible participants at our medical centre. Data from submitted surveys were temporarily stored on the Survey Monkey and iSURVEY websites. At study completion, data were downloaded onto the password-protected computer of the primary investigator (MP) and combined into a single data set for analysis. IBM SPSS statistics, Version 20 (IBM, Armonk, New York, USA) was used to perform all statistical analyses. Participant characteristics and survey data are summarized using mean (standard deviation) for continuous variables and count (percent) for categorical variables. Fisher's Exact Test (two-tailed) was used to assess for between group differences in proportions. In all cases significance was determined at the p<0.05 level.

## Results

The survey response rate was 72% (83/115), with 83% (68/82) self-identifying as nursing professionals and 61% (50/82) as health care providers working in the PCCU setting. Seventy-six percent (63/80) of respondents reported that they worked full time and 56% (47/83) described themselves as experienced or very experienced in performing pediatric resuscitation. Additional details of participant characteristics are listed in Table 1.

**Table 1:** Participant Characteristics.

Survey Item	Response Option	Pediatric	Pediatric	p-value
		Critical Care	Emergency	
		Unit Practice	Department	
		Location	Practice	
		N (%)	Location	
			N (%)	
Clinical Role	Nurse	42 (84)	26 (81)	0.771
	Staff Physician or	8 (16)	6 (19)	
	Subspecialty			
	Trainee			
Work Status	Full Time Staff	43 (88)	20 (65)	0.023
	Part Time or	6 (12)	11 (35)	
	Occasional Staff			
Years of Work Experience in	<2 years	9 (18)	10 (31)	0.080
Pediatric Critical Care or	2 to 5 years	11 (22)	12 (37.5)	
Pediatric Emergency	5 to 9 years	9 (18)	4 (12.5)	
	10 or more years	21 (42)	6 (19)	
Pediatric Resuscitation	None or Minimal	7 (14)	5 (16)	0.202
Experience	Some	10 (20)	13 (40)	
	Experienced	21 (42)	9 (28)	
	Very Experienced	12 (24)	5 (16)	
How frequently do you	Rarely/Almost	7 (14)	10 (31)	0.305
perform emergent fluid	Never			
resuscitation to treat shock	Approximately 1 in	29 (58)	15 (47)	
	10 days/shifts I			
	work			
	Approximately 1 in	6 (12)	5 (16)	
	5 days/shifts I			
	work			
	Approximately half	5 (10)	1 (3)	
	of the days/shifts I			
	work			
	Almost every	3 (6)	1 (3)	
	day/shift I am			
	working			

Fisher's Exact test for comparison of proportions, 2-sided p-value reported

Fifty-eight percent (46/80) of respondents were able to correctly identify that the ACCM pediatric and neonatal septic shock resuscitation guideline requires the administration of 20 mL/kg of isotonic fluid within 5 minutes. Study participants endorsed varying beliefs regarding how to optimally perform pediatric fluid resuscitation for children in shock (Tables 2 and 3). Opinions also differed regarding the factors most important in determining the mode of pediatric fluid resuscitation selected by providers (Table 4).

**Table 2:** Respondent perceptions regarding pediatric fluid resuscitation practices.

Survey Item	Response Option	Nurse	Staff Physician	p-value
		N (%)	or Subspecialty	
			Trainee	
			N (%)	
Fluid resuscitation method	Regular Infusion	8 (12)	0	<0.001
MOST LIKELY to permit	Pump			
you to administer 20	Syringes in	17 (25)	6 (43)	
mL/kg of intravenous	Sequence			
fluid as a bolus to a 2 year	Single Syringe	43 (63)	3 (21)	
old WITHIN 5	(Push-Pull			
MINUTES.	method)			
	Pressure Bag	0	1 (7)	
	Rapid Infuser	0	4 (29)	
	Device e.g.			
	Level 1 Rapid			
	Infuser			
Fluid resuscitation method	Regular Infusion	45 (66)	10 (72)	0.400
LEAST LIKELY to	Pump			
permit you to administer	Syringes in	1 (1)	1 (7)	
20 mL/kg of intravenous	Sequence			
fluid as a bolus to a 2 year	Single Syringe	2 (3)	1 (7)	
old WITHIN 5 MINUTES	(Push-Pull			
	method)			
	Pressure Bag	10 (15)	1 (7)	
	Rapid Infuser	10 (15)	1 (7)	
	Device e.g.			
	Level 1 Rapid			
	Infuser			
Do you feel that you have	No	30 (45)	12 (86)	0.005
received adequate	Yes	37 (55)	2 (14)	
training/education				
regarding the appropriate				
use of the Level 1 Rapid				
Infuser in children				
What is the smallest	≥5 kg	1 (1)	4 (29)	<0.001
weight of child in whom	≥10 kg	5 (7)	3 (21)	
you consider it safe and	≥15 kg	6 (9)	3 (21)	
appropriate to use a Rapid	≥20 kg	36 (53)	4 (29)	
Infuser Device, such as	≥40 kg	19 (28)	0	
the Level 1 Rapid Infuser	≥70 kg	1 (1)	0	
when managing shock				

Fisher's Exact test used for comparison of proportions, 2-sided p-value reported.

**Table 3:** Optimal method of performing emergent fluid resuscitation for shock according to age category.

Fluid Administration	Neonates	Infant/Toddler	Young Child	Older Child
Method	(<1 month)	(1–36 months)	(3–8 years)	(9–17 years)
	N (%)	N (%)	N (%)	N (%)
Regular Infusion Pump	11 (13)	10 (12)	10 (12)	6 (7)
Syringes in Sequence	37 (45)	26 (31)	17 (20)	5 (6)
Single Syringe	35 (42)	46 (55)	41 (49)	12 (14)
(push-pull method)				
Pressure Bag	0	2 (2)	6 (7)	7 (8)
Rapid Infuser Device	0	3 (4)	12 (14)	61 (73)
Unsure which is the	2 (2)	1 (1)	4 (5)	1 (1)
optimal method				
Total Responses (N)	85	88	92	91
Respondents	83	83	83	83
Completing Question				

**Table 4:** Factors determining the fluid resuscitation method selected by health care providers when managing a pediatric patient in shock.

Factor	Respondents selecting
	N (%)
Availability of equipment	27 (33)
Availability of staff	19 (23)
Evidence in the medical/scientific literature	8 (10)
AHA PALS guidelines	25 (30)
Personal preference	3 (4)

82 of 83 participants answered the question, ‘Which of the following is most important in determining your choice of intravenous fluid resuscitation method in a situation where emergent fluid resuscitation is required for shock’

Less than half of those surveyed (47%; 39/82) believed that they had received adequate training/education regarding use of the Level 1 rapid infuser device in children. Differences of opinion also existed regarding the appropriate threshold patient size for use of this device, with physician respondents considering use in smaller patients more acceptable than nurses (p<0.001). Most respondents cited infrequent use of rapid infuser devices in their clinical practice (Table 5). Specific concerns cited by participants in relation to rapid infuser device use in children are listed in Tables 6 and 7.

**Table 5:** Frequency of Level 1 rapid infuser device use among pediatric health care professionals working in emergency department and critical care settings.

Frequency of rapid infuser device use	Respondents selecting
	N (%)
More frequently than once per month	2 (2)
Once per month	12 (15)
Once or twice each year	35 (43)
Once every five year	17 (21)
Never	16 (19)

82 of 83 participants answered the question, ‘How often do you estimate you use a Rapid Infuser Device, such as the Level 1 Rapid Infuser, in your clinical practice?’

**Table 6:** Participant endorsed concerns related to use of rapid infuser devices, such as the Level 1 Rapid Infuser in Children.

Potential Concern	Nurse	Staff Physician or	p-value
	N (%)	Subspecialty Trainee	
		N (%)	
I have no concerns regarding	9 (13)	4 (29)	0.222
the use of Rapid Infuser			
Devices e.g. Level 1 Rapid			
Infuser in Children			
Risk of Air Embolism	39 (57)	4 (29)	0.077
Risk of Pulmonary Edema	20 (29)	5 (36)	0.752
Inadequate time to reassess	14 (21)	1 (7)	0.448
the patient between 20 mL/kg			
fluid boluses			
Other Concern (list)	16 (24)	5 (36)	0.335

Fisher's Exact test used for comparison of proportions, with 2-sided p-value reported. See Table 7 for ‘other concerns’ – specified as free text by participants.

**Table 7:** Participant cited concerns related to use of rapid infuser devices in children.

Participant	Profession	Concern listed
1	RN	inability to give small volumes to small patients
10	MD	Lack of famiarity (mine and RN staff)
13	RN	iv may be too small to hand rapid rate.
14	RN	do not use it frequently enough to feel comfortable with it
16	MD	No knowledge
21	RN	if delivering <500 ml, unsur how to measure appropr.
23	RN	lack of education and lack of use by practitoners
24	RN	staff confidence in using level 1
26	RN	no expeience with rapid infuser
27	RN	delay in time to prime the tubing and trouble shoot errors, we don't use
		it often enough to prime it quickly, or have dedicated staff to use it
31	RN	need for 1∶1 nurse with pump
34	RN	Not able to have 1 to 1 infuser staffing
35	RN	Education
36	RN	blowing the vein with resulting interstitial fluid
41	RN	accuracy of specified volume prescription delivery
43	RN	difficult to admin volumes less than 250/500/1000 ml
45	RN	infrequent usskill = poor skill set
46	RN	we don't use it often enough to feel confident with it.
49	MD	Too large and too fast for small children and infants.
50	RN	not having enough staff who can quickly set up and monitor the device
52	RN	STAFFING, TAKES ONE DEDICATED RN TO RUN
		EFFECTIVELY
53	MD	ability of vascular access to withstand flow rates from infuser
55	RN	INEXPERIENCE
56	RN	administration site concerns
70	RN	staff errors due to infrequency of use
71	RN	we use this so little the time it would take to set up we could have
		administer the fluid already by any other method

Comments reported exactly as provided by participants. RN – Registered Nurse; MD – Physician.

## Discussion

This study of PED and PCCU health care providers reveals differing attitudes and beliefs regarding optimal pediatric fluid resuscitation performance in the non-operative setting. Our findings are of potential importance in light of data indicating a frequent failure to meet recommended fluid resuscitation benchmarks for pediatric shock management in clinical practice. [8], [18], [19] Lack of timely fluid therapy negatively impacts on survival odds [7]–[9] and increasing adherence to existing guidelines may help to improve outcomes. Understanding provider perspectives represents an important first step towards identifying and addressing knowledge gaps and barriers.

### Fluid Resuscitation Preferences differ by patient age

While use of different fluid administration strategies in patients of differing ages/sizes may seem intuitive, respondents in our study still disagreed when presented with discrete patient age categories. In an in vivo study comparing the ability of gravity, push-pull, and pressure bag fluid administration techniques to meet the ACCM guideline goal of 20 mL/kg within 5 minutes, no technique succeeded in patients weighing 40 kg or more. [26] Gravity flow was inadequate regardless of patient weight, pressure bag was variably successful in patients under 40 kg, and the push-pull technique met the ACCM goal in all but one patient under 40 kg who also had a 22-gauge IV catheter in place. Consistent with these findings, respondents in our study cited manual fluid resuscitation methods as being preferred for patients up to 8 years of age.

Our finding that factors independent of patient age/size appear to contribute to practice variation warrants further exploration. A lack of clear direction in current guidelines as to how fluid administration is best performed likely reflects the limited evidence available and this may well contribute to the variable preferences reported. Given that there appears to be room for improvement in fluid resuscitation performance in the clinical setting, further research to support development of evidence-based guidelines defining best practices seems sensible. Future guidelines should acknowledge patient size as important when choosing a fluid administration method.

### Fluid Resuscitation Preferences differ by provider role

It is interesting to note the differences of opinion between physician and nursing staff regarding optimal fluid resuscitation performance. This finding suggests a possible disconnect between theory and practice that may apply to the performance of other resuscitative tasks as well. While physicians may have a good understanding of resuscitation requirements and endpoints, important knowledge concerning the practical aspects of implementation may be lacking. An illustrative example from our study relates to the perceived utility of rapid infuser devices. While physicians may have knowledge of the technical capabilities of these devices, nurses likely have a better understanding of the required setup time, personnel requirements, and operating complexities which may impact treatment initiation.

### Providers are uncertain of the role of rapid infuser devices in pediatric fluid resuscitation

Rapid infuser devices, such as the Level 1, are sophisticated medical devices that can be used to infuse isotonic fluids and/or blood products at high flow rates of up to 500 mL/min. This renders these devices extremely useful in the resuscitation of adult-size trauma patients where the administration of litres of fluid may be required to rapidly restore circulatory volume. The role of rapid infuser devices in the resuscitation of pediatric patients is less clear, and the responses received from study participants reflect this uncertainty.

Providers expressed differences of opinion regarding the threshold age/size of patients in whom use of rapid infuser devices should be considered, with physicians more open to using these devices in small patients. The limited weight-based data available from the Stoner study [26] would suggest that use of rapid infuser devices should be considered for patients weighing 40 kg or more, although other fluid administration techniques may be effective when more than one IV access site is available. At our pediatric tertiary care centre, Level 1 rapid infuser devices are located and ready for use in both the PED and the PCCU. This device is used periodically during both pediatric resuscitations and ‘mock codes’ at the discretion of the responsible physician.

In keeping with known complications that may occur with rapid infusion, [30]–[32] respondents indicated concern about the potential occurrence of adverse events in their patients. Participants also reported differing frequencies of use of rapid infuser devices in the clinical setting, with infrequent use commonly cited. Low frequency of use combined with provider concerns related to knowledge upkeep indicate a need for ongoing in-servicing of staff. Physicians may also benefit from education regarding device setup time, operating complexities, and personnel requirements.

### Practical considerations may be most important in driving provider fluid resuscitation choices

More than half of the respondents in our study cited practical considerations as being most important in determining the mode of pediatric fluid resuscitation selected. In the operative environment, anesthetists can typically anticipate and prepare for the management of acute hypovolemia. In contrast, the non-operative environment is relatively less controlled and less predictable. Settings such as the PED and PCCU must therefore be broadly prepared to manage patients of all ages/sizes. Despite this, equipment may be subject to less frequent use, its location may change, and availability is dependent upon the adequate stocking of supplies. In other hospital areas, non-tertiary settings, and the pre-hospital setting, equipment availability may be even further limited. [33]–[35] The number of providers available to assist with resuscitation and their level of experience is also likely less consistent in the non-operative setting. Current resuscitation guidelines may list a variety of options for performing fluid resuscitation recognizing that what works best in one setting may be impractical another.

### Study Limitations and Strengths

As a single centre study, the main limitation to note relates to the sample size, which limits study power and may impact on the generalizability of our results. Our study also focuses on fluid administration practices used to address acute circulatory instability and does not delve into ongoing controversies regarding the most appropriate fluid type [36], [37] or volume to administer beyond the initial resuscitative phase. [38], [39] Study strengths include rigorous survey development and use of a staged protocol to maximize response rate. Our results suggest a need for further study of the practical aspects of fluid resuscitation performance to determine how health care providers can best achieve recommended benchmarks.

## Conclusions

Differences of opinion regarding how best to perform pediatric fluid resuscitation exist among healthcare providers most likely to resuscitate children in the non-operative setting. Further study is warranted to define best practices and to improve guideline translation into the clinical setting. The role of rapid infuser devices in the resuscitation of pediatric shock requires further clarification.
